# The Progress of Cancer Research: The Need for International Co-Operation

**Published:** 1913-08-09

**Authors:** 


### THE PROGRESS OF CANCER RESEARCH.

### The Need for International Co-operation.

We have on more than one occasion commented on the reports issued by the Imperial Cancer Research Fund, and have expressed the opinion that considerably more valuable data might be derived from systematic investigation of clinical evidence. Hitherto overmuch attention has been devoted to pathological and theoretical investigation, with the result that the literature has been overburdened with a mass of irrelevant deductions which must be sadly embarrassing to future students. This year's report, which was presented on Thursday last week, chronicles a step in the right direction. From it we note that considerable attention has been paid to systematic analysis of cancer statistics. Such investigative analysis has for a long time been pursued by the Germans in connection with their Colonial Department, and it is to be hoped that some co-operation may be secured between the English Fund and German investigators in order to avoid overlapping and economise energy.

### Cancer Houses.

Some attention has been paid to the question of cancer houses. Medical men are well aware of the discussion which was aroused some years ago when some original-minded person enunciated the view that certain residences were "cancer infected" and had an unusually high cancer mortality. Mr. Clement Lucas, we believe, still supports this view, and doubtless there are many medical men who have facts from their experience which induce them to regard the hypothesis with favour. The Research Fund, however, has come to the conclusion that there is very little in it. The question is, after all, a statistical one, and few of us know how to deal with statistics. Indeed, one of the best things that the Fund has done so far is to point out how uncertain and inconclusive statistics with regard to the mortality from cancer are. Thus the cancer death-rates in 1910 are calculated as "occurring in a population with fewer individuals in the age groups over thirty-five than must actually have been the case in the light of the Census of the ensuing year. The cancer rate is, in consequence, inflated by being reckoned on male and female populations with too few members of the age groups which contribute the majority of the deaths from the disease. . . . Analogous considerations serve to minimise the importance of differences in the death-rates from cancer in restricted areas (*e.g.* counties) in any year or series of years. The proportion of the two sexes and of the several age groups for each sex vary from one locality to another. The mortality from all causes varies greatly at different ages and in the case of cancer also for sex, the female sex being more liable. Thus while the death-rate from cancer per million living in England and Wales in 1910 was 856 for males and 1,070 for females, these average rates are exceeded in some counties and not attained in others."

### A Cancer Census.

There are many other interesting points raised in the report. Among these is the desirability or otherwise of instituting a cancer census and of making the disease notifiable. German experience, as summarised by so able an authority as Professor Dr. Kirchner—whose views are quoted in the report—is distinctly against adopting such a course, and until some certain method of diagnosis is discovered it seems hopeless to expect any uniformity in notification. There are many who hold that a certain fallacy is introduced into vital statistics by the notification of even such essentially specific diseases as diphtheria and plague, while it is well known that little reliance can be placed on the statistics in regard to tuberculosis derived from notification. At present we have no test which can be applied to any given case to find out whether or not a certain lesion which is not amenable to microscopical examination is cancerous. Research in this direction would be welcomed; since it seems hopeless to expect a "cancer cure" from laboratory methods, in view of the many failures during the last few years, some of the energy expended on synthetic combinations may justifiably be used to attempt the discovery of a testing serum. The objections to the cancer census are pointed out in the report; they seem to us to be convincing, at least in the present state of our knowledge of the disease.

### Cancer Cures.

Naturally considerable—and let us add gratifying—attention has been devoted to the investigation of so-called cancer cures. Particulars of some of these are given. For the most part they are of the "wild cat" order of things, empirical remedies adopted on utterly inadequate presuppositions of their efficacy. In quite another category stand the recent and highly commendable investigations of certain Continental clinicians who have tried to find combinations of metallic compounds which have a selective destructive action on the cancer cell. The most promising of these is mesothorium, with which experiments are at present being conducted at Düsseldorf. Mesothorium is obtained from thorium earth, as a by-product in the extraction of radium salts; it is at present an enormously expensive remedy, costing many thousands of pounds per gramme, and the amount available for experimental purposes is barely sufficient for a couple of patients. Yet notwithstanding the expense involved it seems that there is reason to hope that this combination does possess some selective action upon the cancer cell. Other vaunted cures have miserably failed, as the violet leaves, the Doyen serum, and the American serum have failed. These failures are discouraging, but it is only fair and reasonable that the claims of the various remedies alleged to be cures shall be fully tested. In this direction the Fund does excellent work, which is mainly, though not entirely, conducted at the Cancer Wing of the Middlesex Hospital.

Wanted, An International Cancer Bureau.

In the future the investigators may justifiably consider some of the native remedies which are applied to tumours in semi-tropical countries. A small commission sent out to investigate cancer incidence in some of the British Colonies overseas will, we feel sure, do good work in this direction, while the expenses in connection with it will be relatively small in proportion to the utility of the commission itself. The public has shown itself fairly generous in supporting the Fund in the past, and will most likely continue to support it in the future, for the report has aroused general interest and has been ably summarised in some of the more influential organs of the lay press. The establishment of a central international cancer bureau would be a welcome sign of the fact that co-operation is active between the various groups that are now, somewhat independently, dealing with matters that are practically identical. Is it too late in the day to hope that a portion at least of the Lister Fund may be used to establish and endow such a central bureau? Surely no greater problem confronts the profession at the present moment than this problem of the treatment of cancer, and every effort should be made to deal with it in an up-to-date and thoroughly modern spirit.

